# Lived Experiences of Patients Hospitalized With Acute Decompensated Heart Failure and Kidney Dysfunction

**DOI:** 10.1001/jamanetworkopen.2024.55008

**Published:** 2025-01-17

**Authors:** Gwen M. Bernacki, Ann M. O’Hare, Mahlet Assefa, Kevin D. O’Brien, David K. Prince, James N. Kirkpatrick, Nisha Bansal, Catherine R. Butler

**Affiliations:** 1 Department of Medicine, University of Washington, Seattle; 2 Cambia Palliative Care Center of Excellence, University of Washington, Seattle; 3 Hospital and Specialty Medicine Service, VA Puget Sound Health Care System, Seattle, Washington; 4 Geriatric Research, Education and Clinical Center, VA Puget Sound Health Care System, Seattle, Washington; 5 Kidney Research Institute, University of Washington, Seattle; 6 Seattle-Denver Center of Innovation for Veteran Centered and Value Driven Care, VA Puget Sound Health Care System, Seattle, Washington; 7 UW Medicine Heart Institute and UW Medicine Diabetes Institute, Seattle, Washington; 8 Department of Bioethics and Humanities, University of Washington, Seattle

## Abstract

**Importance:**

For patients hospitalized with acute decompensated heart failure (ADHF), the presence of kidney dysfunction can substantially shape prognosis and treatment options. Yet little is known about the lived experiences of these medically vulnerable patients.

**Objective:**

To elicit accounts of the illness and care experiences of patients currently or recently hospitalized with ADHF and kidney dysfunction in order to identify potential opportunities to improve care.

**Design, Setting, and Participants:**

This qualitative study of patients with ADHF and kidney dysfunction was conducted at 3 hospitals within an academic health system serving the Greater Seattle area between February 2022 and February 2023. Patients were recruited from those enrolled in a parent study of patients hospitalized with ADHF. A sample of patients who had either acute kidney injury and/or chronic kidney disease were invited to participate in 1 or more qualitative interviews during hospitalization or approximately 3 months postdischarge.

**Main Outcomes and Measures:**

Transcribed interviews were analyzed using inductive thematic analysis.

**Results:**

A total of 40 interviews were conducted among 28 participants. The mean (SD) participant age was 67 (12) years, and 8 (28%) identified as female, 4 as Black [14%], and 20 as White [71.4%]. Qualitative analysis resulted in 4 dominant themes: (1) pervasive impact of functional limitation, (2) adapting goals and changing expectations, (3) struggling to interpret clinical information, and (4) decisional roles and relationships with clinicians. Participants described how their illness had shaped their day-to-day function, life space, and sense of self. They did not always speak in explicit terms about prognosis but most recognized limits on longevity and life options and had started to rethink their priorities. Most participants were engaged in a process of trying to understand the medical aspects of their illness and care, often drawing on their own embodied experience of illness. They had particular difficulty grasping the significance of kidney dysfunction and connecting this to the symptoms they were experiencing. Participants did not always feel equipped or empowered to engage in clinical decision-making, tending to trust the clinicians caring for them.

**Conclusions and Relevance:**

Interviews with adults currently or recently hospitalized with ADHF and kidney dysfunction highlight the importance of improving education, communication, and engagement in care among members of this medically vulnerable group.

## Key Points

**Question:**

What are the lived experiences of patients with acute decompensated heart failure and kidney dysfunction?

**Findings:**

Participants in this qualitative study described how illness shaped their ability to function, sense of self, and future expectations. However, these patients often struggled to understand and fully participate in the medical aspects of their illness and care.

**Meaning:**

The experiences of these patients signal a need and opportunity to improve education, communication, and engagement in clinical care among members of this medically vulnerable population.

## Teaser

This qualitative study examines patient experiences during and after hospitalization with acute decompensated heart failure and kidney dysfunction.

## Introduction

Hospitalization for acute decompensated heart failure (ADHF) is often a sentinel event portending a poor prognosis both during hospital admission and after discharge.^1,2,3,4^ Kidney dysfunction—either in the form of acute kidney injury (AKI) or chronic kidney disease (CKD)—is present in an estimated 30% to 60% of adults with ADHF or chronic heart failure (HF).^5,6,7,8^ In this context, the presence of kidney dysfunction reflects more severe multi-organ disease and can also affect the relative benefits and harms of different HF therapies. For patients with both ADHF and kidney dysfunction, illness trajectories tend to be unpredictable, prognosis is often poor, and clinical care can be extremely complex.^9,10,11^ As the number of potential treatment options for HF continues to expand, patients and their family members are increasingly faced with decisions about treatments for which the benefits may be limited or uncertain.^5,8,12,13,14,15,16^

Little is known about the illness and care experiences of adults with ADHF and kidney dysfunction, both during and after hospitalization. A better understanding of the illness experiences of members of the medically vulnerable group may be helpful in guiding clinical care, education, communication, and process improvement.^17,18,19,20^ To inform efforts to improve care for patients with ADHF and kidney dysfunction, we conducted a qualitative study to elicit the lived experiences and perspectives of a sample of patients with kidney dysfunction participating in an ongoing observational study of patients with ADHF.

## Methods

### Parent Study

The Kidney Injury and Heart Failure (KIND-HF) study is an ongoing prospective study of English-speaking adults aged 18 years or more with ADHF admitted to 1 of 3 hospitals affiliated with the University of Washington (UW) in Seattle, Washington. KIND-HF recruitment sites include a safety net hospital, a quaternary care center, and a community hospital and thus serve a diverse patient population. Exclusion criteria for KIND-HF include the presence of a left ventricular assist device or active receipt of acute or chronic dialysis. Participants are queried about their age, sex, and race and ethnicity at the time of recruitment to the parent study. Race was categorized as American Indian or Alaskan Native, Asian, Black, White, Native Hawaiian or Pacific Islander, 1 or more, or other race. Ethnicity was categorized as Hispanic or Non-Hispanic. Self-reported race and ethnicity data were collected to provide context on possible social determinants of health and the lived experiences of patients in the study. Comoribidity and laboratory values were obtained from the electronic health record. As of February 2023, 128 patients had been recruited into the KIND-HF study.

### Qualitative Substudy

Between February 2022 and February 2023, we invited a sample of participants in the KIND-HF study who also had kidney dysfunction to participate in 1 or more semi-structured interviews to elicit their experiences of illness and care. Kidney dysfunction was defined as having an acute kidney injury (AKI) (serum creatinine levels 0.3 mg/dL or greater above than their most recent preadmission value) or having stage IIIB-V chronic kidney disease (CKD [to convert to millimoles per liter, multiply by 88.4]) (estimated glomerular filtration rate [eGFR] levels less than 45 mL/min/1.73 m^2^ for at least 3 months prior to hospitalization). Participants were recruited and interviewed during their index ADHF hospitalization and/or at their follow-up study visit that occurred at approximately 3 months after discharge. All participants provided written informed consent to participate in the qualitative substudy after discussing what the study would involve and how study findings would be disseminated. The study was approved by the UW Medicine institutional review board.

### Interviews

Four team members (G.M.B., A.M.O., N.B., and C.R.B.) developed an interview guide to elicit participants’ experiences of illness and care with input from other study investigators (eAppendix in Supplement 1). The interview guide was iteratively refined over the course of the study to allow for elaboration of emerging themes. Inpatient interviews were conducted in person and outpatient interviews were conducted by telephone, videoconference, or in-person depending on participant preference, to allow for flexibility in timing and location. Interviews were conducted by 1 of 3 trained research staff members. Interviews lasted approximately 30 to 45 minutes and were audio-recorded with participants’ permission and transcribed verbatim.

### Qualitative Analysis

Deidentified transcripts were analyzed inductively by 4 team members using the constant comparative method to identify dominant themes pertaining to study participants’ lived experiences of illness and care.^21,22^ All transcripts were coded by a cardiologist experienced in qualitative research (G.M.B.) and at least one other member of an analytic team that included a nephrologist experienced in qualitative research (A.M.O.), a nephrologist and KIND-HF principal investigator (N.B.), and a nephrologist and bioethicist experienced in qualitative research (C.R.B.). We used Atlas.ti version 8 (Lumivero) to support the analytic process. The study team met regularly to discuss emerging themes and subthemes, clarify meaning, resolve any differences in interpretation, and select exemplar quotations, returning as needed to interview transcripts and recordings. We continued to recruit and interview study participants until we were confident that we had reached thematic saturation, the point at which dimensions of themes were richly elaborated and stable when tested against new transcripts.^23,24,25^ We analyzed all interview transcripts as a collective body of text rather than seeking to compare themes emerging from particular subsets of transcripts (eg, initial vs follow-up interviews, inpatient vs outpatient contexts) or across demographic groups. All coauthors, who collectively represent expertise in nephrology, cardiology, palliative care, and bioethics, reviewed successive manuscript drafts to provide input on thematic organization and content and to assist with selection of exemplar quotations. Our approach to qualitative analysis and study design was guided by relevant portions of the Consolidated Criteria for Reporting Qualitative Research (COREQ) reporting guideline.

## Results

We recruited a sample of 28 KIND-HF participants who had kidney dysfunction during the index hospitalization for ADHF and conducted interviews during this index hospitalization and/or during a 3-month postdischarge study visit. The 28 participants completed 40 interviews total. Of the 28 participants, 17 had 1 interview, 10 had 2 interviews, 1 had 3 interviews. A total of 10 participants completed both an inpatient and outpatient interview; 23 interviews were conducted during the index hospitalization (median [IQR] time to first interview after admission, 2 [1-3] days) and 17 interviews were conducted after discharge from the index hospitalization (median [IQR] time to first interview after discharge, 87 [74-184] days). The mean (SD) age of participants was 67 (12) years, 8 participants (29%) identified as female; 1 participant (4%) identified as American Indian or Native Alaskan, 1 (4%) as Asian, 4 (14%) as Black, 20 (82%) as White, and 2 (7%) as more than 1 or other race (Table). At the time of the index hospitalization, 27 (96%) had CKD and 25 (89%) had AKI. Based on their most recent echocardiogram, 13 participants (46%) had a reduced left ventricular ejection fraction (LVEF) of 40% or below, 2 had a mildly reduced LVEF (41% to 49%), 9 had a preserved LVEF (50% or higher), and 4 participants could not be classified. Eight participants (29%) died within a year of the index admission date.

**Table.** Participant Characteristics

Characteristics	Participants (N = 28), No. (%)
Age, mean (SD), y	67 (12)
Sex	
Female	8 (29)
Male	20 (71)
Other sex	0
Race	
American Indian or Native Alaskan	1 (4)
Asian	1 (4)
Black	4 (14)
White	20 (71)
Native Hawaiian or Pacific Islander	0
>1 or Other race	2 (7)
Ethnicity	
Hispanic	1 (4)
Non-Hispanic	27 (96)
Index hospital admission site	
Transplantation facility	15 (54)
Community hospital	10 (36)
County hospital	3 (11)
History of CKD at hospital admission	27 (96)
AKI at hospital admission	25 (89)
Serum creatinine at admission, mg/dL	
Mean (SD)	2.2 (0.8)
Median (IQR) [range]	2 (2-3) [1-5]
eGFR, mL/min/1.73 m^2^	
Mean (SD)	35 (14)
Median (IQR) [range]	33 (26-42) [13-71]
Most recent LVEF prior to admission	
Reduced (≤40%)	13 (46)
Mildly reduced (41%-49%)	2 (7)
Preserved (≥50%)	9 (32)
Not available	4 (14)
Died within 12 mos of admission, No. (%)	8 (29)

Abbreviations: AKI, acute kidney injury; CKD, chronic kidney disease; eGFR, estimated glomerular filtration rate; LVEF, left ventricular ejection fraction.

SI conversion factor: To convert creatinine to micromoles per liter, multiply by 88.4.

In qualitative analysis, we identified 4 themes describing participants’ lived experiences of illness and care: (1) pervasive impact of functional limitation; (2) adapting goals and changing expectations; (3) struggling to interpret clinical information; and (4) decisional roles and relationships with clinicians.

### Pervasive Impact of Functional Limitation

Study participants described a range of different symptoms, which were often framed in terms of their impact on everyday function and engagement in life activities (Box 1). For example, one participant described not being able to “breathe deep enough to blow my nose.” Difficulty performing everyday activities could cause frustration and a sense of powerlessness. One participant explained, “It’s one thing to choose not to do [what you want to do]; it’s another not to have that choice anymore.” Life space could also become severely restricted, as one participant explained: “your world becomes whatever is out the window of the room that you’re in.” Many participants were engaged in a process of adapting to their symptoms and impairments by doing less and/or taking more time to accomplish tasks. As one participant explained, “It’s gotten to where I just basically…get to work [stopping often to breathe on the way] and…keep myself fed.”

Box 1. **Example Quotations for Pervasive Impact of Functional Limitation**

I can’t breathe deep enough to blow my nose. (Participant 1)
The biggest thing is, like I said, not being able to do what you want to do. It’s one thing to choose not to do it, it’s another one to not have that choice anymore. (Participant 3)
Your world becomes whatever is out the window of the room that you’re in. (Participant 5)
It’s gotten to where I just basically, you know, all I can do is…to get to work and get home, and that’s about it. Keep myself fed. (Participant 4)
I don’t have the stamina that I normally brought to larger tasks such as building bookshelves for a friend.…There’s been this dissociative split between the person I was formerly and what I am now. So, I feel like I haven’t fully adapted to my new limitations yet. (Participant 6)
I didn’t like just lying there in bed, or sitting in a chair watching TV…. I needed to at least get up and try and feel human. (Participant 5)
I like to cook, so I keep busy cooking. Mostly, I take care of other people. That’s one of the things I missed when I got sick. I drive a lot of people to doctor appointments, take them to the store sometimes, when they need to go shopping. And it’s not so brilliant really. When you think about it, most people have a need to feel needed. Is that a simple way of putting it?…Because I think the one thing that scares me most is to wake up and not have any reason to do something. As stupid as it sounds, it’s a real possibility. (Participant 8)

Changes in what patients could do could sometimes jeopardize their sense of self. As one participant reflected, “[There is a] dissociative split between the person I was formerly, and what I am now.” Another commented: “I didn’t like just lying there...I needed to get up and try and feel human.” Some also described an impact on social relationships. As one participant who was no longer able to help friends with daily tasks reflected, “Most people have a need to feel needed.”

### Adapting Goals and Changing Expectations

Some participants made explicit mention of prognosis: “I asked [the doctor] how much longer [he] thought I had to live” (Box 2). However, most spoke in more general terms about how their health was deteriorating and their time might be limited. Many seemed aware that they had entered a new phase in their illness trajectory during which their health was more precarious and no longer something they took for granted. As one participant explained, “You go to sleep every night not knowing if you’re going to wake up.”

Box 2. **Example Quotations for Adapting Goals and Changing Expectations**

I asked him how much longer [he] thought I had to live, and he said, I can’t answer that for you. Which is understandable. But what I was looking for was answers…But he was real good about explaining it.…Nobody can tell you how long you actually have to live. (Participant 19)
I can’t walk through the store without feeling like I’m going to fall over dead.…I don’t even know what to say, I just don’t want to die. Not yet, I’m too young. I want to go see people that I haven’t seen, my relatives. So, I’m going to do what I can to survive because I don’t want to die yet. It’s scary. Especially when you go to sleep every night not knowing if you’re going to wake up. It’s quite frightening. But I just have to accept God’s will, what happens at this point in time. But if I can do something to change that, then that’s what I’m going to do. That’s why I’m here. (Participant 9)
Dialysis makes you look at your life…all the things I shouldn’t have done. (Participant 11)
We’ve all got a limited time on this earth, so maybe this is the slap in the head for me, that I need to start worrying about that more. And when you start looking at cases that get as complex as mine is, then you start realizing that, well gee whiz, I may not be able to do this, I may not be able to take this path. (Participant 12)
It made my whole situation telescoped. I no longer had the three-and-a-half years to go out there and do the last things that I wanted to do. (Participant 10)

For some, deteriorating health could prompt reflection on life goals and priorities: “Dialysis makes you look at your life…things I shouldn’t have done.” Others tried to make sense of what was happening to them. As one patient said, “We’ve all got limited time on this earth, so maybe this is a slap in the head for me, that I need to start worrying about that more.” Many had become more focused on short-term goals as their life became “telescoped.”

### Struggling to Interpret Clinical Information

Many participants were engaged in an ongoing process of trying to make sense of what they had been told by clinicians and of the care they were receiving (Box 3). It was especially challenging to internalize clinical information when this did not seem to align with patients’ own bodily experience of illness. For example, one participant reflected, “I don’t feel anything in my heart, I’m not breathing heavy.” Some described strategies to learn more about their condition such as listening in on bedside teaching with clinical trainees and doing their own research: “[The doctor is] usually pretty technical, but I’d look it up later.”

Box 3. **Example Quotations for Struggling to Interpret Clinical Information**

My heart? I don’t feel anything in my heart, I’m not breathing heavy. (Participant 14)

[The doctor is] usually pretty technical, but I’d look it up later and figure out what he meant. (Participant 21)
[The kidneys] seem to be doing better, according to the machines. I guess. They’re putting out a lot of urine. (Participant 7)
Well, we haven’t gotten to kidney dialysis yet. So, I guess I’ll deal with that when that happens. My bloodwork shows that something is haywire....I don’t know what it means. (Participant 20)
I mean I’m worried about [the kidneys], obviously, a little bit. And I want it under control. But I can’t feel anything. So it’s not forefront on my mind. Obviously, I don’t want it to get worse, I don’t want it to get to where we have to do something else.…I didn’t really have any symptoms for the kidneys; it was just the heart. (Participant 14)
You’re not as sick as the numbers say you are. I have no problems. I urinate on a regular basis without any struggle.…Let’s look at the patient before we look at the statistics. (Participant 8)

Grasping the meaning and significance of kidney dysfunction could be especially challenging. Patients often viewed urine output as a kind of barometer for how their kidneys were functioning. As one participant commented, “[The kidneys] seem to be doing better.…They’re putting out a lot of urine.” Another participant equated kidney dysfunction with dialysis and commented, “We haven’t gotten to kidney dialysis yet. So, I guess I’ll deal with that when that happens.” However, the patients with whom we spoke tended to attribute the symptoms they were experiencing to HF rather than to kidney dysfunction. For example, one participant commented, “I didn’t really have any symptoms for the kidneys; it was just the heart.” Most understood the importance of laboratory results in the assessment of kidney function and might have a general sense of how things were going based on what they had been told by clinicians, but often lacked a detailed understanding: “My bloodwork shows that something is haywire....I don’t know what it means.” However, a numbers-driven approach that downplayed patients’ bodily experience of illness did not always sit well with patients: “You’re not as sick as the numbers say you are. I have no problems.…Let’s look at the patient before we look at the statistics.”

### Decisional Roles and Relationships With Clinicians

Some patients felt that clinicians were not attuned to what was most important or concerning to them (Box 4). Reflecting on an interaction he had with a member of his clinical team, one participant shared: “Just getting from the waiting room to this room and I’m out of breath, and you want me to exercise?” Another noticed how little the clinical team had to offer when it came to addressing his functional limitations. “[I need] to gain more strength, mostly in my legs. In case I fall again, I need to be able to get up….Nobody seems to have the answer!” Some patients made explicit reference to clinicians’ limited understanding of their illness experience. As one participant explained, “You [the doctor] don’t know anything—this is my body. You can’t know other people…unless you’ve walked in their shoes.”

Box 4. **Example Quotations for Decisional Roles and Relationships With Clinicians**

Just getting from the waiting room to this room and I’m out of breath, and you want me to exercise?...How in the hell do you exercise when your heart’s working twenty-five percent? (Participant 13)
[Interviewer: What are your expectations for the remainder of your hospitalization?] Just to gain more strength, mostly in my legs, in case I fall again I need to be able to get up. And I’m not sure, nobody seems to have an answer. Like I need something, like a jack or something, to get me up from the floor. (Participant 7)
They let you talk. You don’t over talk each other. We’re respectful to each other, yeah. I don’t like doctors that come in like they know it all, they know this, they know that. You don’t know anything. This is my body. You can’t know other people, because you don’t…unless you’ve walked in their shoes, you know? (Participant 18)
I don’t need to know the details: I just need to feel better. (Participant 9)
Well, you know, I have a very good relationship with my cardiologist. We kind of go over everything before everything is done, so I understand. A lot of times I defer to him, because, I mean, he knows more than me. Now, when I was in here 5 weeks ago he called me on the phone, I sent him a text that I was in here and he called me. He said, I want you to look at CardioMed, go online and research it. So I did, and I saw him right after I got out of the hospital, and we discussed it. He said, so do you want to get it? And I said, yeah, let’s do it. I have that scheduled. (Participant 14)
I said, ‘Why can’t I just get off the bed?’ He said, ‘Well it’s dangerous.’…It didn’t matter what I thought.…That was like a slap in the face…but I was happy to be alive, so I tried to just put everything in perspective and just shut up. (Participant 8)
My attitude was, I’m going to be the nicest and most pleasant patient as I can be, because how I go is how they go, and if I make it miserable, they can make it miserable for me. It’s a 2-way street. (Participant 15)

Recognizing that they themselves lacked needed clinical expertise, most patients tended to entrust their care to clinical teams. As one participant commented, “I don’t need to know the details, I just need to feel better.” In reflecting on his discharge planning, one patient commented, “I feel ready to go, but I would defer to [the doctors] if they thought I should stay.” Nonetheless, a few of the patients with whom we spoke described playing a more active role in clinical decision-making: “[We] go over everything before everything is done…[the cardiologist said] go online and research it.’ So, I did…and we discussed it.”

In addition to differences in medical knowledge and expertise, comments from some patients hinted at their limited power in relationships with clinical teams. Gratitude for the care they were receiving often came with a sense of obligation to comply with clinical recommendations. For example, one participant described disagreeing with precautions that kept them restricted to bed: “It didn’t matter what I thought.…That was like a slap in the face…but I was happy to be alive, so I tried to just put everything in perspective and just shut up.” While study participants often lacked a detailed understanding of the power dynamics governing their care, they recognized the need to maintain positive relationships with clinical teams. As one patient commented, “My attitude was, I’m going to be the…most pleasant patient I can be…if I make it miserable, they can make it miserable for me.”

## Discussion

This qualitative study of patients currently or recently hospitalized with ADHF and kidney dysfunction offers a window on the lived experiences of members of this medically vulnerable group. Study participants offered vivid accounts of how their illness had impacted their day-to-day lives, sense of self, life priorities, and hopes and expectations for the future. However, many lacked a detailed understanding of the medical aspects of their illness and did not always feel equipped or empowered to actively participate in their care.

Our findings suggest that there may be untapped opportunities to improve the quality of communication for patients with ADHF and kidney dysfunction. This insight aligns with recent heart failure guidelines^26^ calling attention to the importance of engaging patients in clinical decision-making across the illness spectrum, clarifying goals of care with high-quality communication, increasing access to supportive care, and involving care partners in shared decision-making.^27^ Many patients with whom we spoke had developed an intuitive understanding of their health challenges based on their bodily experience of illness and had already started to rethink life goals and expectations. While most wanted to learn more about their care and prognosis, they often did not feel equipped or empowered to participate in treatment decisions, nor did clinicians always seem attuned to their most pressing concerns.

Our findings also highlight some of the challenges of helping patients to understand the significance of concurrent heart and kidney dysfunction. While separate communication tools have been developed to support decision-making for people with ADHF^28,29^ and for those with CKD,^30,31,32^ prognosis is more guarded and treatment options more limited for those with both conditions. Interestingly, the patients with whom we spoke tended to attribute many of their symptoms and functional limitations to their HF, while the effects of kidney dysfunction seemed less palpable and more difficult for patients to understand. We suspect that patient’s limited understanding of the combined effects of heart and kidney dysfunction probably contributed to their limited engagement and sense of powerless in shaping their clinical care.

These findings underscore a need to improve education for patients with ADHF and kidney dysfunction and enhance communication with the clinicians caring for them. Interviews with patients enrolled in our study perhaps highlight the potential value of a more active and open-ended approach to listening in helping clinicians to gain deeper appreciation for patients’ lived experience both inside and outside health care settings. In particular, a traditional approach to communicating about patient medical history may miss important impacts on physical and social function, independence, and quality of life.^27,33,34^

### Limitations

This study had several limitations. In interpreting our findings, it is important to keep in mind that this study was designed to capture the experiences of patients hospitalized with ADHF and AKI and/or CKD and thus may not be transferable to patients with stable HF, with or without CKD. Furthermore, study participants were recruited from a single academic health system in the Greater Seattle area, which may limit the generalizability of our findings to patients with ADHF and kidney dysfunction more broadly. Furthermore, most participants were White and male, which may limit the transferability of our findings to other groups. Our study was intended to capture the experiences of patients currently or recently hospitalized, but does not support specific comparisons between participant perspectives in different contexts (eg, initial vs follow-up interviews, inpatient vs outpatient setting).

## Conclusions

Our findings describing the lived experience of patients currently or recently hospitalized with ADHF and kidney dysfunction signal both a need and an opportunity to improve education, communication, and engagement in clinical care for members of this medically vulnerable population. Such efforts should be grounded in an understanding of patients’ lived experiences of illness and care.

## Notes

Supplement 1. **eAppendix.** KIND-HF Ethics Supplement—Semi-Structured Interview Guide

Supplement 2.
Data Sharing Statement
