# Supplementary material for: Lived Experiences of Patients Hospitalized With Acute Decompensated Heart Failure and Kidney Dysfunction
Source: JAMA Netw Open. 2025 Jan 17;8(1):e2455008. doi: 10.1001/jamanetworkopen.2024.55008 (PMC11742534; doi:10.1001/jamanetworkopen.2024.55008)
Supplement: Supplement 1. — eAppendix. KIND-HF Ethics Supplement—Semi-Structured Interview Guide [file jamanetwopen-e2455008-s001.pdf]

Supplemental Online Content

Bernacki GM, O'Hare AM, Assefa M, et al. Lived experiences of patients hospitalized with acute decompensated heart failure and kidney dysfunction. *JAMA Netw Open.* 2025;8(1):e2455008. doi:10.1001/jamanetworkopen.2024.55008

eAppendix. KIND-HF Ethics Supplement—Semi-Structured Interview Guide

This supplemental material has been provided by the authors to give readers additional information about their work.

© 2025 Bernacki GM et al. *JAMA Network Open*.

eAppendix. KIND-HF Ethics supplement—Semi-structured interview guide

---

Introduction script (inpatient and clinic visits)

Thank you for being willing to talk with me today. I'm [introduce yourself]. As you know, we are conducting a study to better understand how we care, and can better care, for people with illnesses like yours. We want to learn about your experiences and thoughts about your health and the care you have received. There are no right or wrong answers. I have a list of questions that I will use as a guide to ask about your illness experience and I might have some follow up questions to make sure I understand what you are telling me.

We would like to audio-record interviews so that we can pay full attention to what it is you are saying during the interview rather than trying to write everything down. I might also take some notes to help me remember what you are saying so that I can circle back with any questions. We will not be sharing your responses with your care team and the audio-recording would not be shared with anyone outside the study team.

Do you have any questions about the plan?

We typically spend 30-45 minutes for interviews but that depends on your availability. How much time do you have to talk?

As a reminder, you can stop the interview at any time. You are also free to skip any questions you prefer not to answer, and everything that you say during the interview will remain confidential.

I am part of a research study that is not related to your clinical care. None of what you say will be shared with any of the doctors, nurses, or other staff taking care of you. With this in mind, would you still like to participate in the interview? Would it be OK with you if we audio-record your interview?

[If participant declines] No problem, we completely understand and thank you for considering
[If participant accepts] Great! Thank you for contributing to this project. I'll start the audio recording now if you're ready to begin.

Initial interview guide questions:

Generic probes that can be used with any questions:

- What was that **like**?
- Can you tell me more about [***word*** *that the participant used*]?
- Can you give me an **example**?
- How did that go? What went **well**? What was **hard**?
- Tell me a bit **more**.
- Was anything surprising or **unexpected**?
- What has been **similar or different** than in the past?

1. **Can you tell me about your health conditions?**
   Optional probe: What has it been like to take care of your health?
   [NB: Try to probe for detail based on participants' own telling of their story, but if not mentioned after this has been fleshed out, ok to use these specific probes or come back to them at the end.]
   Do you have any heart problems? [if yes] can you tell me about these?
   Do you have any kidney problems? [if yes] can you tell me about these?

© 2025 Bernacki GM et al. *JAMA Network Open*.

2. **Can you tell you tell us about your experience leading up to your hospital admission and how things have been going for you since you arrived?**

3. **a. Can you tell me about any decisions you (or your family, your doctor, or others on your care team) have had to make about doing a procedure or surgery?**

Optional Probe: For example, in some situations, people with very advanced kidney or heart failure have to decide about dialysis or Ventricular Assist Device (or VAD) treatments.

b. What has been hard about these decisions (and why)?
c. What has been easy about these decisions (and why)?

Optional probes: How do you think about these decisions? Are there other people who help you make care decisions (for example, family, friends, care team members)? How do they help you?

4. **What is important about your life and the way that you live your life now?**

*Optional Probe*s: What matters most to you?
Do any key goals come to mind (e.g., things you want to do or are important that you continue doing)?

5. **How do you expect things will go in the future?**

Optional Probes: What do you hope for?
What worries you?

6. **We've talked through many of the things that I had planned to ask you about. Is there anything we have not covered that you would you like to bring up?**

Closing script:
Thank you again for taking the time to talk with me and for participating in this research. We hope that this will help us take better care of other people like you.

Follow-up interview guide questions:

Generic probes that can be used with any questions:

- What was that **like**?
- Can you tell me more about [***word** that the participant used*]?
- Can you give me an **example**?
- How did that go? What went **well**? What was **hard**?
- Tell me a bit **more**.
- Was anything surprising or **unexpected**?
- What has been **similar or different** than in the past?

1. **Can you tell me about your health and the care that you have been receiving?**

Optional probes: What kinds of health problems have you had? What is it like to take care of your health?
[NB: Try to probe for detail based on participants' own telling of their story, but if not mentioned after this has been fleshed out, ok to use these specific probes or come back to them at the end.]

Have you had any other health conditions?
Do you have any heart problems? [if yes] can you tell me about these?
Do you have any kidney problems? [if yes] can you tell me about these?

2. **We want to understand better what it is like to have an illness like yours. Would you be able to tell us a little more about what it has been like for you these past few months?**

© 2025 Bernacki GM et al. *JAMA Network Open*.

3. **Can you tell me about interactions with health care personnel (for example, nurses, doctors, therapists, or others) over the past year?**
   Optional probes: Who have you discussed your health problems with? How did your care providers explain things to you? What did that mean to you?

4. **Have you had to make any decisions about your health or treatment options? What has that been like?**
   Optional probes: How do you make these decisions?

5. **Are there other people who help you make care decisions (e.g., family, friends, care team members)? How do they help you?**

6. **We've talked through many of the things that I had planned to ask you about. Is there anything we have not covered that you would you like to bring up?**

Closing script:
Thank you again for taking the time to talk with me and for participating in this research. We hope that this will help us take better care of other people like you.

© 2025 Bernacki GM et al. *JAMA Network Open*.
