# Supplementary material for: Lived Experiences of Patients Hospitalized With Acute Decompensated Heart Failure and Kidney Dysfunction
Source: JAMA Netw Open. 2025 Jan 17;8(1):e2455008. doi: 10.1001/jamanetworkopen.2024.55008 (PMC11742534; doi:10.1001/jamanetworkopen.2024.55008)
Supplement: Supplement 2. — Data Sharing Statement [file jamanetwopen-e2455008-s002.pdf]

Data Sharing Statement

Bernacki. Lived Experiences of Patients Hospitalized With Acute Decompensated Heart Failure and Kidney Dysfunction. *JAMA Netw Open*. Published January 17, 2025.
doi:10.1001/jamanetworkopen.2024.55008

Data
Data available: Yes
Data types: Deidentified participant data
How to access data: email to nbansal@uw.edu
When available: With publication

Supporting Documents
Document types: Statistical/analytic code
How to access documents: email to nbansal@uw.edu
When available: With publication

Additional Information
Who can access the data: researchers with an approved proposal
Types of analyses: specified purpose
Mechanisms of data availability: after approval of proposal
